# Ectopic Intrauterine Device Revealed by Ureteral Colic in a 37-Week Pregnant Woman: Case Report

**DOI:** 10.3390/healthcare10061060

**Published:** 2022-06-08

**Authors:** Alexandra Matei, Mihai Cornel Traian Dimitriu, Irina Pacu, Crîngu Ionescu

**Affiliations:** Department of Obstetrics and Gynecology, Carol Davila University of Medicine and Pharmacy, 050474 Bucharest, Romania; drmihaidimitriu@yahoo.com (M.C.T.D.); irinapacu@hotmail.com (I.P.); antoniuginec@yahoo.com (C.I.)

**Keywords:** copper IUD, pregnancy, ureteral colic, omentum

## Abstract

Copper T intrauterine devices (IUDs) are a popular long-acting reversible contraceptive method. The most common reasons for contraceptive failure are expulsion and extrauterine migration. We report a case of a 28-year-old female, G13P3, 37 weeks pregnant, who presented to the hospital for left abdominal flank pain. The patient was admitted for treatment of left ureteral colic. The woman went into labour, and Caesarean Section was performed due to foetal distress. During the surgery, an inspection of the peritoneal cavity revealed a copper IUD embedded in the granulous tissue located in the left lateral abdominal region, which was extracted. No uterine scar tissue could be identified macroscopically. The migration of an IUD in the abdominal cavity is a rare finding, and coexistence with third-trimester pregnancy is an infrequent but serious event due to potential visceral complications. Higher gravidity can be associated with an increased risk of IUD migration in women with a non-scarred uterus.

## 1. Introduction

Worldwide, intrauterine devices (IUDs) are the second most popular form of contraception, after female sterilization [1]. According to Population Reference Bureau, in 2019, 6.3% of Romanian women aged 15 to 49 years old were using IUDs for contraceptive purposes [2]. Among the long-acting reversible contraceptive (LARC) methods that currently exist, copper-based IUDs are approved for up to 10 years of use, managing to prevent fertilization through its highly effective spermicidal mechanism of action and inhospitable environment for implantation [3,4]. This is the outcome of a marked local foreign body reaction determined by the IUD adequate placement, characterized by a significant increase in the number of neutrophils, mononuclear cells and plasma cells [5]. Additionally, the release of copper ions is responsible for the endometrial mucosa becoming unfavourable for implantation by interfering with the local enzymatic and proliferative activity and for the inhibition of fertilization secondary to both alterations in the biochemical composition of the fluid inside the uterine cavity and impairment of sperm viability [5,6].

The contraceptive failure rate for copper IUDs is one of the highest among LARC methods, ranging from 0.1 to 2.2/100 women years [7]. The contraceptive effectiveness of LARCs is independent of parity and age, but studies have shown that the main variables associated with copper IUD perforations were breastfeeding (RR: 4.9, 95% CI: 3.0–7.8) and time since delivery (RR: 3.0, 95% CI: 1.5–5.4) [8].

Although commonly used worldwide and well tolerated by most patients, the insertion of the device consists of a procedure with inherent risks of complications such as displacement outside the uterine cavity in the cervical canal or embedment in the myometrium and perforation of the uterus [9]. Expulsion along with migration of the IUD from its normal positioning in the uterine fundus are also responsible for decreases in its contraceptive efficacy; complete uterine perforation in which the device is partially or completely within the peritoneal cavity assumes careful evaluation of intraabdominal lesions, requiring surgical management [4]. Large studies confirm that most perforations are either asymptomatic or associated with mild symptoms such as abnormal bleeding, mild pain or both, combined with unintended pregnancy [1,7]. Despite 85% of reported cases of asymptomatic perforations at the time of diagnosis, there are certain medical situations when clinical examination can identify fever, abdominal pain, diarrhoea, urinary tract infection, occlusive syndrome or peritonitis as serious complications of IUD perforation [10,11].

The aim of this paper is to report a case of a third trimester pregnancy complicated with ureteral colic in which a copper-based IUD was identified in the peritoneal cavity during Caesarean section and to discuss the existing literature data regarding this peculiar context.

## 2. Case Report

**Figure 1:**
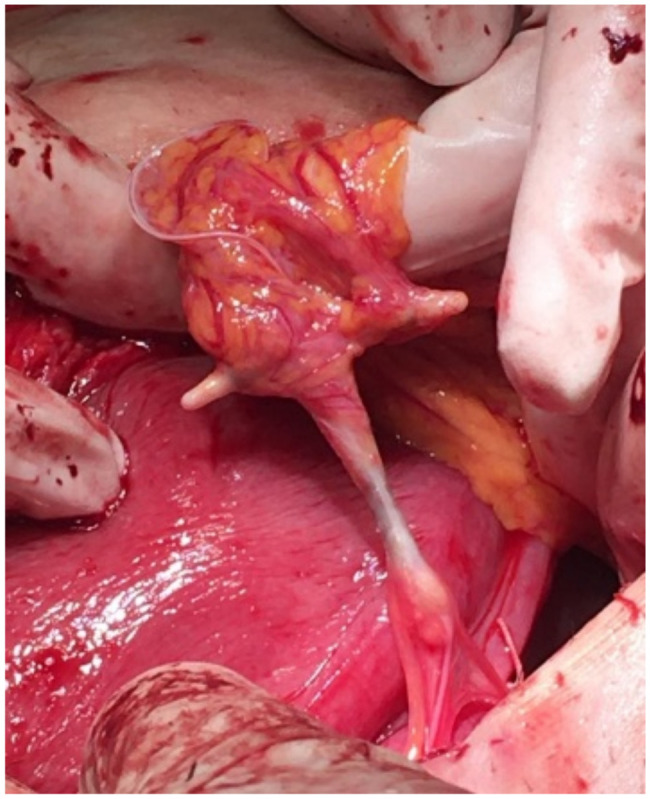
Intraoperative visualization of the ectopic localization of the IUD in the peritoneal cavity.

**Figure 2:**
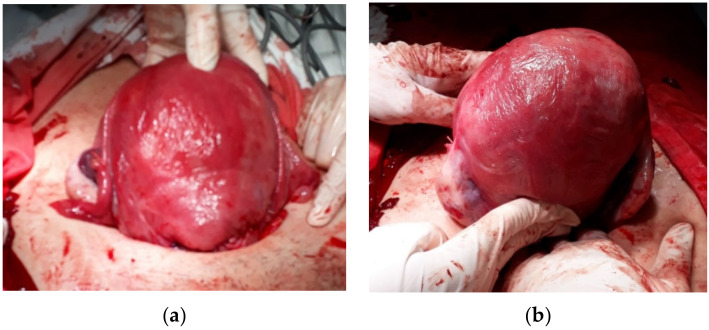
No associated pathological aspects suggestive for perforation on the (**a**) anterior uterine wall, and (**b**) fundal region and posterior uterine wall.

A 28-year-old female, G13P3, was referred to the present medical institution during the third trimester of pregnancy for left abdominal flank pain. At the time of referral, she had 37 weeks of amenorrhea and no prior follow-up pregnancy visits. The obstetrical history involved 2 previous vaginal deliveries at term and 10 uncomplicated abortions by dilation and curettage. The patient was admitted for treatment of left ureteral colic and further investigations on the pregnancy. The clinical and paraclinical assessment confirmed the diagnostic suspicion of a urinary tract infection complicating a left ureteric calculus. Specific antibiotic and symptomatic therapy were initiated. An obstetrical evaluation identified a high-risk pregnancy with oligohydramnios and estimated foetal weight at the 2.5th percentile, suggestive for intrauterine growth restriction. The patient went into spontaneous labour, and Caesarean section was performed due to foetal distress. The inspection of the peritoneal cavity during surgery revealed a copper IUD embedded in the granulation tissue, located in the left lateral abdominal region, adherent to the large omentum (Figure 1). The device was removed along with part of the omentum associated with chronic inflammatory infiltration and was sent for histopathological examination. An accurate evaluation of the superior genital tract was conducted for signs of perforation. There were no pathological findings on the uterine walls and the adnexal regions (Figure 2a,b).

The woman recalled an IUD placement 2 years prior to the moment of the current admission without any particular events associated; she had no corresponding follow-up consults, and since she became pregnant, she considered that the IUD was expulsed spontaneously.

The postoperative evolution of the patient was favourable and uneventful. The histopathological report confirmed a benign fibrotic process in the omentum.

## 3. Discussion

The high level of effectiveness regarding IUD contraception associated with good tolerance and safety of the method justify their use regardless of age and parity [12]. Complications related to IUD vary significantly, from vaginal bleeding and pelvic infections to even more rare events such as expulsion, uterine perforation, fragmentation, or infertility [3,13,14].

Most IUD perforations are primary and occur during insertion in 0.4–1.1 per 1000 procedures, while secondary perforations are challenging to identify eight weeks or more after application [9,15,16]. Occurring later after the insertion, the migration of an IUD into the peritoneal cavity as a form of secondary perforation can lead to damage of the internal organs [11].

It has been stated that women experiencing IUD perforations are usually multiparas in their early thirties and that for specific secondary perforations, abnormally arranged myometrial fibres and contractions of the uterus represent contributing factors [9,15]. This is also the case of the woman described above, who was younger than 30 years old and had a relevant obstetrical history with respect to potential disruption of the myometrium architecture.

There is scarce data in the literature regarding perforation occurring years after IUD placing, with the median time for diagnosis being approximately 5 months [15]. In our specific case, the patient recalled an IUD placement 2 years prior to the moment of the current admission but without any exceptional events associated. A possible explanation for the incidental diagnosis of IUD perforation resides in the fact that most of the time, only mild symptoms can be identified during perforation: pelvic pain and abnormal bleeding; the uterine wall defect usually heals by itself, while one third of the patients can be completely asymptomatic—in this context, the diagnosis is established retrospectively, usually secondary to an unintended pregnancy [15,17].

The contraceptive role of IUDs is dependent on its adequate intrauterine positioning. The migration of an IUD in the abdominal cavity is a rare finding, and coexistence with third-trimester pregnancy is an infrequent but serious event due to the potential visceral and obstetrical complications. Previous reports concerning ectopic intraabdominal IUDs concurring with pregnancy development are limited to first and second trimester gestations [18,19,20,21].

In the case of complete perforation of all three layers of the uterus, complications related to adhesion formation, such as infertility, chronic pain and intestinal obstruction, add to potential further perforations of the adjacent structures, causing haemorrhage, fistulas and peritonitis [4]. Approximately 16% of these patients develop intraabdominal infections [4] related to the typical places of migration: adnexa, bladder, broad ligaments, rectosigmoid colon, peritoneum, omentum or the small intestine [16,22]. Previous studies have described a constellation of urinary symptoms in patients with bladder embedded ectopic IUDs: haematuria, urodynia, urinary frequency and urgency, and even renal colic when ureteral compression followed the insertion of a branch of IUD in the ureter [17,23]. However, urinary tract complications are favoured by the pregnancy status due to multiple associated physiological adaptations. Consequently, only in exceptional circumstances with relevant medical history should the identification of urinary symptoms in a pregnant woman imply considering IUD migration as a differential diagnosis.

The presence of a foreign body in the peritoneal cavity can cause damage to vital organs on account of the chronic inflammatory response; chronic abdominal pain can further refer to a wide spectrum of severity, and depending on the migration situs and associated tissue injury, the result can manifest as classic presentation of acute abdomen [9]. In our report, a near-term pregnancy was carried without remarkable clinical events until left abdominal pain symptoms prevailed in the context of a suspected urinary pathology.

Women with implanted IUDs should be examined periodically to verify the device’s positioning [22]. In this particular case the patient had no follow-up after IUD insertion. The presence of the device outside the uterine cavity permitted the implantation and development of an unplanned pregnancy. The time when the dislocation occurred remains unknown as the patient was completely asymptomatic for more than two years following IUD application.

## 4. Conclusions

The present case underlines several interesting points: pregnant women without previous obstetrical check-ups must always have a comprehensive medical history documented while thorough and meticulous clinical and paraclinical examination should be recorded. In some situations, the migration of an IUD outside the uterine cavity can be a clinically silent event; therefore, any association with unintended pregnancy should prompt reassessment of the patient. Increased emphasis should be placed upon non-compliant patients with history of multiple abortions and/or high parity, in whom myometrial architectural disturbances can interfere with the appropriate positioning of the device. Furthermore, an attentive examination of pelvic and abdominal organs must be performed and documented during caesarean section surgery, having in mind the possibility of unexpected findings.

The peculiarity of this case consists of the incidental discovery of a migrated IUD inside the peritoneal cavity during an emergency Caesarean section due to foetal distress performed in a 37-week pregnancy.

## Data Availability

Not applicable.
